# Exploring networks of complex developmental trauma symptomatology among children and adolescents involved in child welfare

**DOI:** 10.1002/jcv2.12224

**Published:** 2024-03-12

**Authors:** Jackson A. Smith, Jasmine Zhang, Alexey Urusov, Laura Colucci, Imogen Sloss, Lillian Eckert, Mary Price‐Cameron, Dillon T. Browne

**Affiliations:** ^1^ Department of Psychology University of Waterloo Waterloo Ontario Canada; ^2^ Centre for Mental Health Research and Treatment University of Waterloo Waterloo Ontario Canada; ^3^ Therapeutic Family Care Program Cobourg Ontario Canada

**Keywords:** adolescents, child welfare, children, complex trauma symptoms, gender differences, network analysis

## Abstract

**Background:**

Clinical presentations of child and adolescent psychopathology can vary systematically for boys and girls. While network analysis is increasingly being applied to explore psychopathology in adults, there is a dearth of network studies considering differences in symptoms for boys and girls, particularly in developmental trauma‐related symptomatology.

**Methods:**

This study involves rural children (*n* = 375, 39.47% girls) and adolescents (*n* = 291, 51.20% girls) involved with child protection services in Ontario, Canada. Caregivers completed the Assessment Checklist for Children or Adolescents within the first 6 months of care. Psychometric network analyses were conducted using subscales for boys and girls. Differences were examined via network comparison permutation tests, moderated network models, and independent *t*‐tests.

**Results:**

Attachment‐related interpersonal difficulties were the most central nodes in the child and adolescent networks for both boys and girls. Emotional dysregulation also had high strength centrality for adolescents. While network comparison tests found the overall network structures and global network strength to be invariant between boys and girls for children and adolescents, moderated network models and independent *t*‐tests revealed several differences with regards to the expression of specific symptoms. Among children, girls exhibited more indiscriminate and pseudomature interpersonal behaviors, whereas boys expressed significantly more non‐reciprocal interpersonal behaviors and self‐injury. Adolescent girls exhibited more behavioral dysregulation and suicide discourse in the moderated network model; *t*‐tests also indicated higher levels of emotional dysregulation, negative self‐image, and other items considered clinically important complex trauma symptoms (e.g., distrust of adults, confused belonging).

**Discussion:**

This study supports evidence of differences in the expression of complex trauma symptomatology for boys and girls. Additionally, girls exhibit more symptoms, in general. Consistent with the transdiagnostic conceptualization of the consequences of developmental trauma, findings demonstrate the primacy of attachment‐specific difficulties and emotion dysregulation.

## Graphical abstract

The present study investigates complex trauma symptom networks among children and adolescents who have experienced maltreatment (i.e., neglect and abuse) and are involved with child welfare in Ontario, Canada. The results support evidence of gender differences in the expression of complex trauma symptomatology among girls and boys across development, including that girls tend to exhibit more symptoms. Findings demonstrate the primacy of attachment‐specific difficulties and emotion dysregulation, consistent with the transdiagnostic conceptualization of the consequences of developmental trauma.

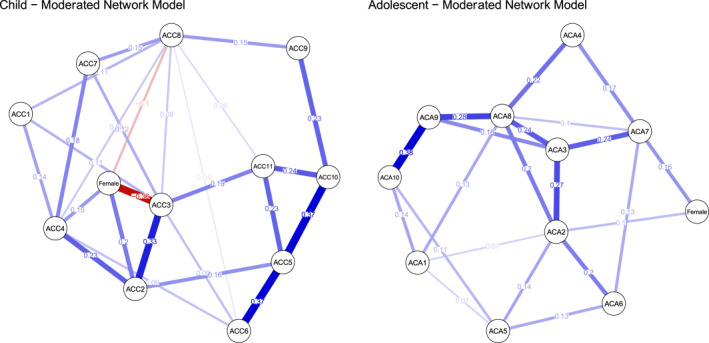

**Key points**

- Previous research indicates differential expressions of complex trauma among boys and girls, but limited work has examined if the interrelationship between symptoms differ.
- Across gender and developmental stage, attachment‐related difficulties were most central.
- Consistent with previous research, the present study observed gender differences in the presentation of complex trauma symptomatology and found that girls, regardless of developmental stage, tend to exhibit more symptoms than boys.
- The structure of complex trauma symptom networks is invariant between boys and girls in childhood and adolescence.
- Clinical assessment and treatment should use validated, developmentally appropriate measures of complex trauma symptomatology, which include measures of attachment security and emotional dysregulation. Further, the centrality of attachment‐specific symptoms and emotional dysregulation indicate that interventions that target attachment security and emotion regulation may be particularly potent.

## INTRODUCTION

Recent perspectives on psychopathology suggest that mental health challenges, including post‐traumatic stress disorder (PTSD), may be best conceptualized as a systemic network wherein symptoms are mutually reinforcing and interdependently influential (Borsboom et al., 2021). To date, several studies have examined PTSD symptom networks in children and adolescents (Bartels et al., 2019; De Haan et al., 2020; Scharpf et al., 2022), but none have specifically explored complex trauma symptoms. Moreover, previous work indicates differences between boys and girls in rates and domains of psychopathology across childhood and adolescence, including trauma‐related symptoms (Hayward & Sanborn, 2002; Hudziak et al., 2007), yet no studies have examined these gender differences from a network perspective. Thus, the present study furthers extant knowledge on complex trauma by investigating differences in symptom networks for boys and girls among children and adolescents who have experienced maltreatment (i.e., neglect and abuse) and are involved with child welfare in Ontario, Canada.

### Complex developmental trauma

The term complex developmental trauma describes both children's exposure to multiple traumatic events and the immediate and long‐term effects of this exposure (Cook et al., 2003). In terms of exposure, complex developmental trauma has been defined as the “…experience of multiple, chronic and prolonged, developmentally adverse traumatic experiences, most often of an interpersonal nature (e.g., sexual or physical abuse, war, community violence) and with early‐life onset” (van der Kolk, 2005, p. 402). Complex developmental trauma yields a wide range of detrimental immediate and long‐term neurodevelopmental and psychosocial impairments, especially when the exposures occur during critical or sensitive developmental periods (Nelson et al., 2019; Nelson & Gabard‐Durnam, 2020). Such experiences initiate a negative developmental cascade that affects biological, psychological, emotional, and social processes over time (Cicchetti & Toth, 2016; D’Andrea et al., 2012; Perry, 2009; Sheridan & McLaughlin, 2020; Toth & Manly, 2018). Relatedly, attachment‐based symptomatology seems most prevalent in the early years, while difficulties related to self‐esteem, self‐concept, substance use, and suicidality become more apparent in adolescence.

Retrospective studies have estimated that the combination of interpersonal victimization and disrupted primary caregiving can explain 45% of the variance in the population attributable risk for childhood‐onset psychopathologies (Green et al., 2010; Norman et al., 2012; Teicher & Samson, 2013). Some have even suggested that individuals with psychopathology and who have a history of childhood maltreatment comprise a clinically and neurobiologically distinct subgroup. Teicher and Samson (2013) argue that individuals in this subgroup have earlier onset, greater symptom severity, more comorbidity, more consistent reductions in hippocampal volume and amygdala hyperreactivity, a greater risk for suicide, and poorer treatment outcomes.

Since Herman (1992) first introduced the term *complex trauma* into the psychiatric lexicon, several studies have attempted to capture the scope of complex developmental trauma sequalae (Cook et al., 2003, van der Kolk, 2005). In these papers, the authors reviewed literature linking complex developmental trauma exposure to several domains of impairment, including attachment disruption, biological and somatic dysregulation, affect regulation, dissociation, issues with behavioral and impulse control, cognitive and attentional impairments, and compromised self‐concept. A review by D’Andrea et al. (2012) subsequently identified symptoms related to affect and behavioral dysregulation, disturbances of attention and consciousness, distortions in attributions, and interpersonal difficulties. In addition, many of the studies reviewed by D’Andrea and colleagues indicated that the expression of symptoms and biopsychosocial impairments tended to occur when children and youth had multiple experiences of interpersonal trauma and should therefore be considered as interrelated symptoms rather than independent, as per the dictates of the current psychiatric nosology.

Children exposed to traumatic experiences exhibit symptom profiles that do not fit internalizing–externalizing dimensions and often do not meet full criteria for PTSD (Ford et al., 2022; Spinazzola et al., 2018). Relatedly, common measures of trauma for children and adolescents fail to capture the unique symptoms expressed by children who have experienced maltreatment, including those involved in out‐of‐home care and child protection services (Denton et al., 2017; Tarren‐Sweeney, 2013a). However, several questionnaires, the majority of which are caregiver‐report measures, have been developed to capture trauma sequalae beyond PTSD symptoms (see Denton et al., 2017 for a review), including the caregiver‐report Assessment Checklist for Children (ACC; Tarren‐Sweeney, 2007) and Adolescents (ACA; Tarren‐Sweeney, 2013b) and the self‐report International Trauma Questionnaire for Children and Adolescents (ITQ‐CA; Cloitre et al., 2018).

Given the complexity of the sequelae described above, individuals with histories of complex developmental trauma are often diagnosed with multiple comorbid disorders, which are viewed as discrete problems to be addressed rather than as facets of one larger problem (Herman, 1992; D’Andrea et al., 2012; Kretschmar et al., 2017). Such multi‐diagnostic formulations pose challenges for children, caregivers, and clinicians when it comes to understanding children's difficulties in the context of their trauma histories, as well as being harder to treat (Nanni et al., 2012; Shenk et al., 2014). This is particularly evident in children involved in child welfare, as they tend to represent the extreme end of the distribution with respect to exposure to abuse and neglect and symptom severity and chronicity (Fisher, 2015; Pace et al., 2019; Tarren‐Sweeney, 2008).

In recognition of complex trauma's unique neurobiological and phenotypic signature, the National Child Traumatic Stress Network developed criteria for a new disorder, *Developmental Trauma Disorder (DTD)*, proposed to be included in the fifth edition of the American Psychiatric Association's (APA) Diagnostic and Statistical Manual of Mental Disorders (DSM‐5). The proposed diagnostic criteria included the following: *Lifetime contemporaneous exposure to interpersonal victimization and primary caregiver attachment disruption* (Criterion A); *Affective and Somatic Dysregulation* (Criterion B); *Attentional and Behavioral Dysregulation* (Criterion C); and *Self and Relational Dysregulation* (Criterion D) (see Ford et al., 2018 for the full criteria). A systematic review by Morelli and Villodas (2021) examined the construct validity and clinical utility of DTD based on the 21 empirical studies (including 17 separate samples) published between 2005 and July 2021. The studies largely supported the DTD construct, including divergent and convergent validity and complex trauma exposure being associated with DTD symptoms. Additionally, factor analytic studies consistently found a three‐factor solution for DTD symptoms measured with a variety of DTD‐specific and ad hoc DTD scales to offer the best fit to the data. However, the review highlights the need for more research with larger samples by research groups who are not associated with the developers of the construct and for the use of more rigorous methodological and analytic designs.

Parallel efforts to those for the DSM, which have aimed to refine the diagnostic precision and clinical utility of the World Health Organization's (WHO) International Classification of Diseases (ICD) classification of trauma responses, led to the formal inclusion of complex PTSD (CPTSD) in the 11th version of the ICD (WHO, 2018). CPTSD is characterized by three symptom clusters related to disturbances in self‐organization (DSO), including negative self‐concept, emotional dysregulation, and disturbances in relationships, in addition to the three PTSD criteria (re‐experiencing, avoidance, and sense of current threat) (WHO, 2023). While the ICD‐11 CPTSD is not specifically a developmental disorder like DTD (i.e., symptom onset can occur in adulthood), several developmental presentations are noted, including a recognition that children and adolescents are more vulnerable than adults to developing CPTSD when exposed to severe or prolonged traumata, neurobiological consequences in children and adolescents can result in cognitive difficulties and executive functioning deficits, distinct behavioral presentations of emotional dysregulation and interpersonal difficulties can be observed among children and adolescents, and that children and adolescents, for whom the source of the trauma was their parent(s) or primary caregiver(s), often develop a disorganized attachment style (WHO, 2023). Factor analytic work has supported the distinction between ICD‐11 PTSD and CPTSD in children and adolescents (Haselgruber et al., 2020; Perkonigg et al., 2016; Sachser et al., 2017).

### Gender differences in (complex) trauma sequalae

A nuanced understanding of developmental psychopathology necessitates examination of gender‐based differences, which are present in general mental health difficulties and trauma‐specific symptoms. The focus of this paper concerns differences for boys and girls, though differences in trauma‐related symptomatology across other genders is an important area of inquiry. In childhood and adolescence, girls tend to exhibit more internalizing psychopathology symptoms, which may increase the use of maladaptive coping strategies (e.g., rumination), create more conflictual social relationships (Martin & Hadwin, 2022), and result in lower life satisfaction (Campbell et al., 2021; Carragher et al., 2016; Yoon et al., 2022). Conversely, boys tend to exhibit higher externalizing challenges and more disruptive behavior (Carragher et al., 2016). A similar pattern is found in trauma‐exposed children and adolescents (Feiring et al., 1999; Gray & Rarick, 2018), though studies with chronically victimized children/youth suggest that girls show higher severity (Ascendio et al., 2022) and a greater variety of symptoms, regardless of the symptom type (Wamser‐Nanney & Cherry, 2018), and are more likely to meet proposed CPTSD diagnostic criteria (Hébert & Amédée, 2020; Redican et al., 2022; Sachser et al., 2017; Solva et al., 2020). Behavioral manifestations of difficulties (e.g., aggression) are more apparent among boys, while sexual related trauma symptoms and self‐esteem problems are more common amongst girls. With that said, not all studies are consistent in finding gender differences with regards to complex trauma responses (Brewin et al., 2017), and more research is needed to determine factors that may influence differential response patterns, including the expressions of specific types of symptoms. Furthermore, most of the existing research that has considered gender differences has focused on average differences in the expression of specific symptoms and rates of meeting diagnostic criteria; consequently, differences in the interrelationships of trauma‐related symptomatology across these groupings remains underexplored. Ongoing research exploring gender‐based differences in trauma symptomatology will enable more specific, client‐centered etiological models, informed by unique patterns of prognosis, prevalence, and symptom profiles (Hartung & Lefler, 2019; Martin & Hadwin, 2022).

### Child and adolescent trauma symptom networks

Network analysis has gained stride as a tool for establishing the empirical foundation of the network perspective of psychopathology, which conceptualizes mental disorders as the product of complex networks of associated symptoms, rather than as the common cause of symptoms (Borsboom, 2017; Fried et al., 2017). The network approach redresses shortcomings of the traditional (common cause) perspective—such as the inability to account for the co‐occurrence of symptoms (Borsboom & Cramer, 2013)—by conceptualizing disorders as a system that arises from symptom interactions, studied in their full complexity without imposed boundary restrictions.

Several recent studies have examined networks of PTSD and comorbid symptoms in children and youth who have experienced trauma and adversity. Using self‐ and caregiver‐report data from an international clinical sample of children and adolescents, Bartels et al. (2019) identified negative cognitions and emotion dysregulation as the most central nodes in self‐report symptom networks; challenges related to re‐experiencing and hypervigilance were additionally central in the caregiver‐report network. De Haan et al. (2020) also investigated PTSD and depression symptoms in an international sample of children and youth, finding re‐experiencing‐related PTSD symptoms and impaired concentration in depression to be the most central. Additionally, Scharpf et al. (2022) examined PTSD symptom networks in a sample of war‐affected children and adolescents from across six countries. Re‐experiencing and avoidance were most strongly connected to other symptoms in the full sample, as well as child‐ and adolescent‐specific networks. These findings collectively illustrate the advantages of network analysis for understanding trauma‐related symptoms. However, the studies relied on PTSD diagnostic criteria, which fail to capture the full breadth of symptoms expressed by children and adolescents who have experienced complex developmental trauma (repeated and/or multiple exposures to interpersonal violence and attachment disruptions; Cook et al., 2005; D’Andrea et al., 2012; van der Kolk, 2005, 2009). Thus, there is a need for network studies to employ measures of complex trauma specifically tailored to such settings when considering symptom profiles among children and adolescents.

### Research questions

The present study examines the interrelationships among complex trauma‐related symptoms in child and adolescent boys and girls living in rural Ontario, Canada, who are involved with child protection services due to substantiated maltreatment. We explored: (RQ1) How are different symptoms of complex trauma interrelated in children and adolescents who have been exposed to interpersonal trauma, (RQ2) Are there gender‐related differences in the structure of complex trauma symptoms amongst children and adolescents, (RQ3) Does the strength centrality of attachment‐related symptoms differ in networks amongst boys and girls, and (RQ4) Do children and adolescents show varying patterns of gender‐related differences in the structure of complex trauma symptoms? Given that network analysis research is exploratory in nature, we did not have specific hypotheses for the structure of the networks. However, we expected girl networks to be denser (i.e., more significant connections between nodes) and with higher strength of conditional associations between symptoms (i.e., higher global strength) and for attachment‐specific and dissociation‐related symptoms to be more influential over the network (i.e., higher strength centrality). We also anticipated behavioral dysregulation symptoms to have higher strength centrality in boy networks.

## METHOD

The preregistration and reproducible code are available at https://doi.org/10.17605/OSF.IO/S9Y6C and https://osf.io/86wdn/, respectively.

### Participants and procedure

Data used in the present study were collected by the Therapeutic Family Care Program (TFCP). TFCP provides therapeutic services to children and adolescents with substantiated cases of maltreatment and their caregivers (biological, kinship, adoptive, and foster caregivers) referred by the Children's Aid Societies of Durham, Kawartha‐Haliburton, and Highland Shores in Ontario, Canada. These catchment areas are large, rural regions falling outside nearby urban centers. Biological or alternative caregivers consented to participate in the agency's services, which included routine measures of children's/youth's psychosocial functioning to monitor treatment progress. Children and adolescents expressed verbal assent for participation. This cross‐sectional analysis includes data from the first assessment for each child/youth, which were collected between 2000 and 2019. Data were available for 555 children, though we included a subset aged 5–11 years (*n* = 375; 39.47% girls; *M*_age_ = 7.6, *SD*_age_ = 2.1) to match the sample used to validate the assessment measure. Data were available for 291 adolescents (51.20% girls; *M*_age_ = 14.2, *SD*_age_ = 1.9). This study was approved by the University of Waterloo Research Ethics Board (ORE #41024) (Table 1).

**TABLE 1:** Demographic characteristics of the full study sample.

	Children (*n* = 375)		Adolescents (*n* = 291)	
	*n*	%	*n*	%
Gender				
Girl	148	39.47%	149	51.20%
Boy	227	60.53%	142	48.80%
Care type				
Birth parent	44	11.70%	44	15.10%
Kinship care	91	24.30%	42	14.40%
Adoption	48	12.80%	34	11.70%
Foster care	186	49.60%	169	58.10%
Group home	6	1.60%	2	0.70%

	*M*	*(SD)*	*M*	*(SD)*
Age (Years)	7.57	2.06	14.16	1.92
Age into care (Years)	4.78^a^	2.84	7.89^a^	4.62

^a^ Age Into Care was provided for *n* = 199 (53.7%) children and *n* = 138 (47.42%) adolescents.

### Measures

#### Assessment Checklist for Children

To assess children's complex trauma symptoms, caregivers completed the Assessment Checklist for Children (ACC), a 120‐item instrument that captures the extent to which children in care exhibit various psychological, social, and behavioral difficulties in response to trauma over the past 4–6‐months (Tarren‐Sweeney, 2007). Caregivers rated each item on a three‐point Likert scale (0 = *Not true* to 2 = *Mostly true*). Items are organized on 10 clinical subscales: (1) *sexual behavior* (11 items; e.g., “Sexual behavior not appropriate for his/her age,” “Sexual relations with an adult”), (2) *pseudomature interpersonal behavior* (8 items; e.g., “Precocious (talks or behaves like an adult),” “Treats you as though you were the child, and s/he was the parent”), (3) *nonreciprocal interpersonal behavior* (12 items; e.g., “Avoids eye contact, except if in ‘trouble,’” “Does not show affection”), (4) *indiscriminate interpersonal behavior* (8 items; e.g., “Attention‐seeking behavior,” “Too friendly with strangers”), (5) *insecure interpersonal behavior* (14 items; e.g., “Fears you will reject him,” “Worries that something bad will happen to you”), (6) *anxious distrustful* (10 items; e.g., “Distrusts adults,” “Is fearful of being harmed”), (7) abnormal pain response (4 items; e.g., “Does not cry,” “Laughs if hurt”), (8) *food maintenance* (4 items; e.g., “Eats too much,” “Hides or stores food”), (9) *self‐injury* (14 items; e.g., “Asks to be physically punished,” “Causes injury to her/himself”), (10) *suicidal discourse* (7 items; e.g., “Attempts suicide,” “Describes how s/he would kill herself/himself”). There is also an *other items* subscale, which represents items that hold clinical significance for children's complex trauma symptoms but did not fit into another scale based on exploratory factor analysis during scale development (Tarren‐Sweeney, 2007, p. 10 items; e.g., “Can't concentrate, short attention span,” “Thinks s/he is someone or something else,” “Has blackouts or periods of amnesia”), and two low self‐esteem scales: *negative self‐image* (9 items; e.g., “Believes s/he is no good at anything,” “Feels worthless or inferior”), and *low confidence* (8 items; e.g., “Does not speak up for her/himself,” “Gives up too easily”). Each subscale score is derived by summing all items, such that higher scores reflect more difficulties. Normative, borderline, and clinical ranges for ACC subscale raw scores can be found in Table S1 in the supplemental file. The ACC shows strong psychometric properties in previous research with children in out‐of‐home care (⍺ = 0.70–0.89; Tarren‐Sweeney, 2014). Cronbach's *⍺* on each ACC subscale in the present study were mostly acceptable or excellent; however, two subscales (Anxious Distrustful and Sexual) were borderline acceptable (both *⍺* = 0.68) and we excluded the Abnormal Pain Response and Other Items subscales due to poor internal reliability (see Table S1 in the supplemental file).

#### Assessment Checklist for Adolescents

Adolescents' complex trauma symptoms were assessed using the Assessment Checklist for Adolescents (ACA; Tarren‐Sweeney, 2013b). The ACA examines a broad range of mental health challenges observed among youth in care aged 12–17 years. Caregivers rated the extent to which adolescents displayed difficulties on 105 items, each rated on a 3‐point Likert scale (0 = *Not true* to 2 = *Mostly true*), over the past 4‐to‐6 months. The ACA included seven clinical scales: (1) *Non‐Reciprocal Interpersonal Behaviors* (10 items; e.g., “Avoids eye contact, except if in ‘trouble,’” "Does not show affection”), (2) *Social Instability/Behavioral Dysregulation* (21 items; e.g., “Impulsive (acts rashly, without thinking),” “Lacks guilt or empathy,” “Relates to strangers as if they were family”), (3) *Emotional Dysregulation/Distorted Social Cognition* (14 items; e.g., “Extreme emotional reaction to minor event (or for no obvious reason),” “Shows intense and inappropriate anger,” “Is convinced that friends will reject him/her”), (4) *Dissociation and Trauma Symptoms* (7 items; e.g., “Nightmares about specific events or people,” “Can't tell if an experience is real or a dream,” “Has panic attacks”), (5) *Food Maintenance* (7 items; e.g., “Eats secretly,” “Hides or stores food”), (6) *Sexual Behaviors* (7 items; e.g., “Forces or pressures other youth or children into sexual acts,” “Inappropriately shows genitals to others,” “Sexual relations with an adult”), (7) *Suicide Discourse* (6 items; e.g., “Attempts suicide,” “Describes how s/he would kill himself/herself”). Additionally, there are two low self‐esteem scales (*Negative Self‐Image* [9 items; e.g., “Dislikes himself/herself,” “Feels ashamed,” “Feels worthless or inferior”], and *Low Confidence* [8 items; e.g., “Does not speak up for her/himself,” “Gives up too easily,” “Lacks confidence”]) and an *Other Items* subscale (15 items). Most items of the *Other Items* subscale relate to insecure attachment (e.g., “clingy,” “seems insecure,” “fears adult rejection”), with the remaining items addressing trauma‐specific responses (e.g., “traumatic memories”, “wary or vigilant”) and maladaptive coping (e.g., “rocks back and forth”, “throws self against walls”). Totals were derived by summing all items on that scale, and higher scores reflect more challenges. Normative, borderline, and clinical ranges for ACA subscale raw scores can be found in Table S1 in the supplemental file. Previous evaluations of the ACA have shown strong psychometric properties based on adolescents in out‐of‐home care (⍺ = 0.76–0.92; Tarren‐Sweeney, 2014). Cronbach's *⍺* in the present study were acceptable to excellent, aside from the Dissociation and Trauma Symptoms subscale (*⍺* = 0.57; see Supplemental Table S2). We chose to retain this scale due to the relevance for the present analyses.

### Analysis

#### Descriptive statistics and outlier management

All analyses were performed in RStudio Version 4.1.2. We examined the distribution and normality of the ACC and ACA by calculating each subscale's mean, standard deviation, skew, and kurtosis using the *psych* package (Revelle, 2022). All analyses were conducted with and without outliers being winsorized to examine the extent of outlier influence. Results presented are with the unmodified data due to the winsorized dataset yielding nearly identical results.

#### Psychometric network analysis

Psychometric network analysis captures the potential inter‐related nature of symptoms (Bringmann et al., 2022), which may give rise to a broader disorder profile (e.g., CPTSD) rather than treating symptoms as being caused by a common CPTSD factor. Thus, instead of assuming that there is a generalized CPTSD factor causing negative self‐concept, emotion dysregulation, interpersonal difficulties, re‐experiencing, avoidance, and a sense of being under threat, a network of these mutually‐reinforcing symptoms could jointly yield a clinical presentation of CPTSD (Fried, 2022; Fried et al., 2017). Taking such an approach can be beneficial for examining meaningful connections among symptoms, lending insight into the nature of how symptoms are interrelated and, consequently, promising treatment targets (Fried et al., 2017; Henry et al., 2022).

We estimated child and adolescent‐specific Gaussian graphical models (GGM; i.e., a network of partial correlations using continuous variables) for boys and girls, as differences in the ACC and ACA precluded the merging of the child and adolescent data. We used the *bootnet* R package (Epskamp et al., 2018) to determine the number of statistically significant edges for all generated networks (*p* < 0.05), as well as the mean edge weight (i.e., average of edge weights in the network). To estimate networks, we applied partial correlation regularization techniques (i.e., least absolute shrinkage and selection operator [LASSO] regularization with the Extended Bayesian Information Criterion (Foygel & Drton, 2010) with a hyperparameter of 0.5) to identify definitive, parsimonious networks. LASSO regularization is a model selection approach that involves testing the fit of a series of models with various edges constrained to zero, in order to determine the model that fits the data best (Tibshirani, 1996).

As recommended by Epskamp et al. (2018), we examined the accuracy and stability of networks via bootstrapping procedures (2500 draws). This enabled examinations of the accuracy of estimated edge‐weights, the stability of node centrality indices, and testing for significant differences between edge‐weights and centralities. The stability of centrality indices was quantified via the CS‐coefficient (Epskamp et al., 2018), which reflects the maximum proportion of cases that can be dropped to retain a correlation with the original centrality of higher than 0.70, with 95% certainty. CS‐coefficients above 0.25 were interpreted (Epskamp et al., 2018). With regards to centrality metrics, we focused on node *strength* (i.e., how influential a symptom is in the network, calculated by summing the absolute edge weights of edges per symptom; see RQ2) as it is of particular relevance for psychological networks (Bringmann et al., 2019), though other common centrality measures, including *closeness*, *betweenness*, and *expected influence*, are reported in the supplemental materials.

We used the *qgraph* (Epskamp et al., 2012) package for network visualizations, which are comprised of nodes (signifying symptoms) and edges (signifying the conditional relationship—partial correlations—between two symptoms). In the GGMs, the distance between nodes as well as edge width and transparency reflect the strength of the association between nodes. Thus, nodes closer together with wider and darker edges have stronger conditional associations. Missing edges indicate a non‐significant partial correlation. Blue and red edges indicate positive and negative associations, respectively.

#### Network comparison test

We compared networks for boys and girls with the *NetworkComparisonTest* package (Van Borkulo et al., 2023). This permutation test statistically compares networks on (i) overall network structure (test statistic = *M*; represents the maximum difference in edge strength of the two networks) and (ii) global network strength (test statistic = *S*; represents the summed value of all the edges in a network) (Van Borkulo et al., 2023). Significant differences in edge strengths between gender networks can be tested post‐hoc for non‐invariant network structures.

#### Symptom severity comparison

In addition to the NCT, which compares network structures and overall network strength, we examined differences in associations between boys and girls with each symptom by including a dummy variable for gender (boy = 0, girl = 1) as a node in moderated network models (Haslbeck, 2022) for child and adolescent symptoms using the *bootnet* package. To examine the degree to which moderation effects emerge with and without controlling for all other effects, we also conducted independent samples *t*‐tests for all reliable ACC and ACA subscales using the *stats* package (R Core Team, 2021). Tests for homogeneity of variance were conducted before computing *t*‐tests. Student's *t*‐tests were conducted for comparisons in which the variances were found to be homogenous; Welch's *t*‐tests were conducted in cases of heteroscedasticity. We corrected for multiple comparisons using the false discovery rate control method (Glickman et al., 2014) to minimize type I error.

## RESULTS

### Descriptive statistics

Table 2 presents the descriptive statistics of the ACC and ACA subscales for children and adolescents, respectively. Based on clinical cut‐offs for each of the subscales, and consistent with this being a clinical sample, most children and adolescents had clinically relevant symptoms, with 86.10% of children and 87.30% of adolescents having at least one clinical scale in the clinical range (Table 3). Children had an average of *M* = 3.02 (SD = 2.05) and adolescents had an average of *M* = 2.84 (*SD* = 1.84) clinical scales above the clinical cut‐off.

**TABLE 2:** Full sample and gender‐specific descriptive statistics of clinical subscales for children and adolescents.

	Full sample		Boys		Girls		*p*	Cohen's d
	*M*	*(SD)*	*M*	*(SD)*	*M*	*(SD)*		
Children – ACC scales								
Anxious‐distrustful	4.53	3.46	4.49	3.59	4.59	3.28		0.03
Food maintenance	1.58	2.01	1.67	2.07	1.45	1.91		0.11
Indiscriminate interpersonal behaviors	7.66	3.56	7.24	3.32	8.31	3.81	*	0.31
Insecure interpersonal behaviors	7.38	5.18	7.06	4.83	7.86	5.66		0.15
Low confidence	6.62	3.69	6.89	3.62	6.22	3.78		0.18
Negative self‐image	4.66	4.62	4.77	4.74	4.5	4.44		0.06
Non‐reciprocal interpersonal behaviors	6.84	4.69	7.56	4.96	5.74	4.03	***	0.40
Pseudomature interpersonal behaviors	4.92	3.73	4.44	3.53	5.65	3.92	*	0.33
Self Injury total	2.59	3.39	2.99	3.73	1.99	2.70	*	0.31
Sexual behaviors	1.29	2.35	1.37	2.48	1.17	2.13		0.09
Suicide discourse	0.76	2.06	0.92	2.34	0.51	1.52		0.20
Adolescents – ACA scales								
Behavioral dysregulation	14.88	8.84	12.89	8.24	16.78	9.01	***	0.45
Dissociation and trauma symptoms	2.00	2.21	1.79	2.14	2.19	2.27		0.18
Emotional dysregulation	10.45	6.73	9.38	6.53	11.46	6.79	*	0.31
Food maintenance	3.29	3.72	3.12	3.75	3.45	3.69		0.09
Low confidence	7.38	3.75	7.19	3.86	7.56	3.63		0.10
Negative self‐image	5.98	4.87	5.04	4.67	6.88	4.91	**	0.38
Non‐reciprocal interpersonal behaviors	6.70	4.23	6.68	4.63	6.71	3.81		0.01
Other items	8.74	5.18	7.82	5.13	9.60	5.10	**	0.35
Sexual behaviors	1.09	2.11	1.16	2.24	1.01	1.99		0.07
Suicide discourse	1.71	2.88	1.01	2.07	2.38	3.35	***	0.49

*Note*: *p*‐values for Students and Welches *t*‐tests: *p < 0.05, **p < 0.01, ***p < 0.001. All *p*‐values were corrected for multiple comparisons using the False Discovery Rate correction.

**TABLE 3:** Range frequencies and percentages of assessment checklist for children (ACC) and assessment checklist for adolescents (ACA) clinical subscales.

Clinical Scales	Boys			Girls		
	Normal	Borderline	Clinical	Normal	Borderline	Clinical
	*n* (%)	*n* (%)	*n* (%)	*n* (%)	*n* (%)	*n* (%)
Children – ACC scales						
Abnormal pain response	199 (87.67%)	13 (5.73%)	15 (6.61%)	139 (93.92%)	8 (5.41%)	1 (0.68%)
Anxious distrustful	86 (37.89%)	45 (19.82%)	96 (42.29%)	60 (40.54%)	33 (22.3%)	55 (37.16%)
Food maintenance	162 (71.37%)	35 (15.42%)	30 (13.22%)	113 (76.35%)	22 (14.86%)	13 (8.78%)
Indiscriminate interpersonal behavior	67 (29.52%)	56 (24.67%)	104 (45.81%)	39 (26.35%)	28 (18.92%)	81 (54.73%)
Insecure interpersonal behavior	60 (26.43%)	40 (17.62%)	127 (55.95%)	40 (27.03%)	16 (10.81%)	92 (62.16%)
Non‐reciprocal interpersonal behavior	54 (23.79%)	37 (16.3%)	136 (59.91%)	40 (27.03%)	25 (16.89%)	83 (56.08%)
Pseudomature interpersonal behavior	86 (37.89%)	44 (19.38%)	97 (42.73%)	40 (27.03%)	27 (18.24%)	81 (54.73%)
Self Injury index	147 (64.76%)	36 (15.86%)	44 (19.38%)	114 (77.03%)	19 (12.84%)	15 (10.14%)
Sexual behaviors	142 (62.56%)	40 (17.62%)	45 (19.82%)	110 (74.32%)	20 (13.51%)	18 (12.16%)
Suicide discourse	177 (77.97%)	N/A	50 (22.03%)	125 (84.46%)	N/A	23 (15.54%)
Adolescents – ACA scales						
Social instability/Behavioral dysregulation	31 (21.83%)	28 (19.72%)	83 (58.45%)	12 (8.05%)	19 (12.75%)	118 (79.19%)
Dissociation/Trauma symptoms	79 (55.63%)	40 (28.17%)	23 (16.2%)	70 (46.98%)	43 (28.86%)	36 (24.16%)
Emotional dysregulation/Distorted social cognition	51 (35.92%)	6 (4.23%)	85 (59.86%)	37 (24.83%)	5 (3.36%)	107 (71.81%)
Food maintenance behavior	105 (73.94%)	12 (8.45%)	25 (17.61%)	102 (68.46%)	17 (11.41%)	30 (20.13%)
Non‐reciprocal	45 (31.69%)	16 (11.27%)	81 (57.04%)	37 (24.83%)	25 (16.78%)	87 (58.39%)
Sexual behaviors	96 (67.61%)	21 (14.79%)	25 (17.61%)	100 (67.11%)	30 (20.13%)	19 (12.75%)
Suicide discourse	102 (71.83%)	N/A	40 (28.17%)	81 (54.36%)	N/A	68 (45.64%)

### Network models: Interrelations among complex trauma symptoms for children and adolescents

#### Network structure

**FIGURE 1:**
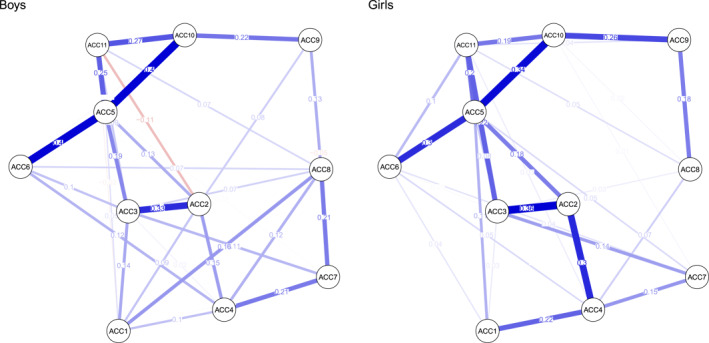
Network of ACC subscales for children: Boys and girls. ACC1: Sexual behaviors. ACC2: Pseudomature interpersonal behaviors. ACC3: Non‐reciprocal interpersonal behaviors. ACC4: Indiscriminate interpersonal behaviors. ACC5: Insecure interpersonal behaviors. ACC6: Anxious‐distrustful. ACC7: Food maintenance. ACC8: Self‐injury. ACC9: Suicide discourse. ACC10: Negative self‐image. ACC11: Low confidence.

The networks of interpersonal trauma symptoms based on the ACC for boys and girls are visualized in Figure 1. Each network included 11 nodes representing scores on each clinical scale. Networks were moderately sparse: of the 55 possible connections, 34 were retained in the boy network (*Ρdensity* = 0.62) and 30 were retained in the girl network (*Ρdensity = *0.55). Mean edge weights (i.e., partial correlation coefficients) for boys and girls were 0.074 and 0.070, respectively. Edge weights ranged from −0.11 (*pseudomature interpersonal behavior—low confidence*) to 0.40 (*insecure interpersonal behavior*—*anxious‐distrustful*, *insecure interpersonal behavior*—*negative self‐image*) in the boy network, and from 0.01 (*non‐reciprocal interpersonal behavior*—*anxious‐distrustful*, *food maintenance*—*negative self‐image*) to 0.36 (*pseudomature interpersonal behaviors*—*non‐reciprocal interpersonal behaviors*) in the girl network.

**FIGURE 2:**
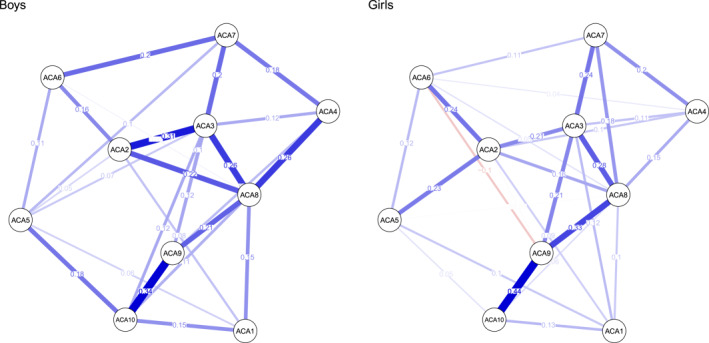
Network of ACA subscales for children: Boys and girls. ACA1: Non‐reciprocal. ACA2: Social instability/behavioral dysregulation. ACA3: Emotional dysregulation/distorted social cognition. ACA4: Dissociation/trauma symptoms. ACA5: Food maintenance. ACA6: Sexual behavior. ACA7: Suicide discourse. ACA8: Other items. ACA9: Negative self‐image. ACA10: Low confidence.

For adolescents, ACA networks (Figure 2) included 10 nodes with 45 possible connections. Of these edges, 26 were retained in the boy network (*Ρdensity* = 0.58) and 29 were retained in the girl network (*Ρdensity* = 0.64), suggesting moderate parsimony. For boys, edge weights ranged from 0.02 (*sexual behavior*—*other items*) to 0.37 (*behavioral dysregulation*—*emotion dysregulation*), with a mean of 0.09. Edge weights in the girl network ranged from −0.10 (*sexual behavior*—*negative self‐image*) to 0.44 (*negative self‐image*—*low confidence*), with a mean of 0.09. Weight matrices for all networks can be found in the supplementary materials (Tables S3 and S4).

#### Node strength

In the child networks, the most central complex trauma symptoms for boys, as indicated by standardized strength estimates, were *insecure interpersonal behaviors* (2.31), *non‐reciprocal interpersonal behaviors* (0.90), *negative self‐image* (0.32), *pseudomature interpersonal behaviors* (0.31), and *indiscriminate interpersonal behaviors* (0.06), respectively; *suicide discourse*, with a value of −1.30, was the least central symptom. For girls, the most central symptoms were *insecure interpersonal behaviors* (2.04), *non‐reciprocal interpersonal behaviors* (0.59), *pseudomature interpersonal behaviors* (0.53), *low confidence* (0.50), *indiscriminate interpersonal behaviors* (0.47), and *negative self‐image* (0.47), respectively; *self‐injury* (−1.23) was the least central symptom.

In the adolescent networks, the most central symptoms for boys were *other items* (1.62), *emotion dysregulation* (1.48), *low confidence* (0.38), *negative self‐image* (0.26), and *behavioral dysregulation* (0.11), respectively; *non‐reciprocal interpersonal behaviors* (−1.61) was the least central symptom. Among adolescent girls, *other items* (1.64), *emotion dysregulation* (1.17), *negative self‐image* (0.90), and *behavioral dysregulation* (0.70) were the most central symptoms; *food maintenance* (−1.10) was the least central symptom. See Table S5 and Figures S1 and S2 in the supplemental materials for all centrality indices.

#### Network stability

Stability analyses indicated that all four networks were accurately estimated, with moderate confidence intervals (CIs) around the edge weights (Figures S3 and S4 in the Supplemental Materials). Most CIs overlapped, implying that edges within networks were unlikely to be significantly different from each other; hence, the order of edge weights should be interpreted with caution. Conversely, the edges for *insecure interpersonal behaviors*—*negative self‐image* (ACC5‐ACC10) and *insecure interpersonal behaviors*—*anxious‐distrustful* (ACC5‐ACC6) are reliably two of the strongest edges in the boy network due to their bootstrapped CIs not overlapping with that of several other edges in the network. Additionally, the edge *negative self‐image*—*low confidence* (ACA9–ACA10) in the network of adolescent girls was reliably stronger than several other edges due to non‐overlapping CIs. The CS‐coefficient for strength centrality for the four networks was 0.59, 0.36, 0.51 and 0.60 for boys and girls in the child and adolescent networks, respectively, exceeding the recommended thresholds for stable estimation (Epskamp et al., 2018). Overall, these metrics indicate that the stability of both edge‐weights and strength centrality indices in the estimated networks were acceptable. Additional details are available in the supplementary materials, including in Table S6.

### Gender differences in complex trauma symptoms

#### Network comparison test

The network comparison test for the child sample revealed that the global strength and structure of the networks for the boys and girls were invariant (*S* = 0.14, *p* = 0.788; *M* = 0.20, *p* = 0.440). Similarly, the networks for adolescent boys and girls were invariant (*S* = 0.08, *p* = 0.772; *M* = 0.19, *p* = 0.645). Due to the invariance in the network structures (i.e., the *M* statistic), we did not proceed with testing invariance in the strength of specific edges in either the child or adolescent samples.

#### Pairwise comparisons

**FIGURE 3:**
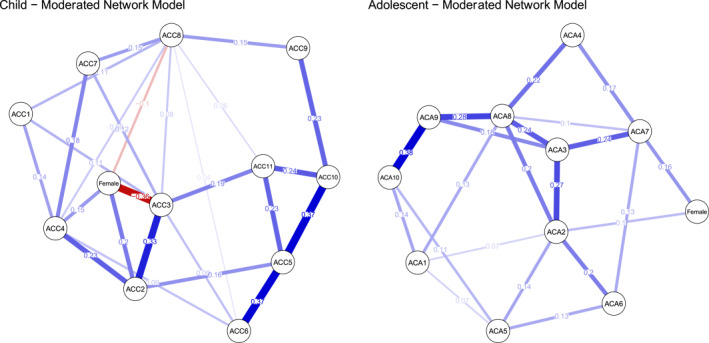
Moderated network models. ACC1: Sexual behaviors. ACC2: Pseudomature interpersonal behaviors. ACC3: Non‐reciprocal interpersonal behaviors. ACC4: Indiscriminate interpersonal behaviors. ACC5: Insecure interpersonal behaviors. ACC6: Anxious‐distrustful. ACC7: Food maintenance. ACC8: Self‐injury. ACC9: Suicide discourse. ACC10: Negative self‐image. ACC11: Low confidence. ACA1: Non‐reciprocal. ACA2: Social instability/behavioral dysregulation. ACA3: Emotional dysregulation/distorted social cognition. ACA4: Dissociation/trauma symptoms. ACA5: Food maintenance. ACA6: Sexual behavior. ACA7: Suicide discourse. ACA8: Other items. ACA9: Negative self‐image. ACA10: Low confidence.

The moderated network model in the child sample (see Figure 3, left panel) revealed that girls expressed significantly more symptoms of *indiscriminate* and *pseudomature interpersonal behaviors*, whereas boys expressed significantly more symptoms of *non‐reciprocal interpersonal behaviors* and *self‐injury*. *T*‐tests in the child data were consistent with the moderated network model (*indiscriminate interpersonal behaviors*: *t* (373) = −2.88, *p* = 0.011; *pseudomature interpersonal behaviors*: *t* (373) = −3.09, *p* = 0.011; *non‐reciprocal interpersonal behaviors*: *t* (355.09) = 3.89, *p* < 0.001; self‐injury: *t* (369.08) = 3.02, *p* = 0.011). The magnitude of effect sizes for pairwise comparisons, as measured by Cohen's d, were small. See Table 2 for respective scale means and effect sizes.

In the adolescent sample, gender significantly interacted with *behavioral dysregulation* and *suicide discourse*, such that girls experienced significantly higher levels of both, after controlling for all other effects (Figure 3, right panel). *T*‐tests were less conservative, finding that girls expressed significantly more symptoms of *behavioral dysregulation* (*t* (289) = −3.84, *p* < 0.001), *emotional dysregulation* (*t* (289) = −2.67, *p* = 0.016), *negative self‐image* (*t* (289) = −3.27, *p* = 0.003), *suicide discourse* (*t* (248.66) = −4.22, *p* < 0.001), and *other items* (*t* (289) = −2.97, *p* = 0.007). The magnitude of effect sizes for pairwise comparisons, as measured by Cohen's d, were small. See Table 2 for respective scale means and effect sizes.

## DISCUSSION

Complex developmental trauma encompasses a range of experiences and symptoms that extend beyond PTSD. Moreover, post‐traumatic stress symptomatology can vary across factors such as age and gender. Building on previous research examining gender differences in children's trauma symptomatology (e.g., Wamser‐Nanney & Cherry, 2018), this is the first network study of differences between boys and girls as assessed by a developmentally sensitive questionnaire that captures the breadth of symptoms expressed by maltreated children and adolescents in out‐of‐home care (Denton et al., 2017). Hence, we aimed to enhance the current understanding of gender differences in complex trauma‐related symptomology and their interrelations through the application of psychometric network analysis.

### Network composition of complex trauma symptomatology

The centrality indices in all networks underscore the primacy of the attachment‐specific symptoms, negative self‐concept, and emotional dysregulation, irrespective of developmental stage and gender. The ACC included four attachment‐specific measures (*indiscriminate*, *insecure*, *non‐reciprocal*, and *pseudomature interpersonal behaviors*) in children. These symptoms had the highest strength centrality in the networks for both boys and girls—especially *insecure interpersonal behaviors*. Additionally, symptoms related to negative self‐identity also emerged as central. While the ACA does not have specific attachment‐related subscales, many of the items in the *Other Items* scale are the same as those from the attachment‐specific scales of the ACC. Consistent with the child networks, this subscale had the highest strength centrality for adolescents, in addition to *emotion dysregulation*, *behavioral dysregulation*, and those related to negative self‐identity (*low confidence* and *negative self‐image*). The centrality of the *emotion dysregulation* and negative self‐concept symptoms is consistent with previous network studies of PTSD symptoms in children and adolescents (Bartels et al., 2019; De Haan et al., 2020; Scharpf et al., 2022). Together with the importance of attachment dysregulation symptoms, these patterns support the transdiagnostic model of developmental mechanisms that explain the link between childhood trauma and psychopathology (McLaughlin et al., 2020) and is consistent with the conceptualization of developmental trauma disorder (DTD; van der Kolk et al., 2009).

### Gender differences in complex trauma symptomatology

This study found that the network structure of complex trauma symptomatology in children and adolescents, as measured by the ACC and ACA, respectively, were invariant across boys and girls. This indicates that interrelationships among complex trauma symptoms are consistent in boys and girls in both childhood and adolescence, lending credence to the conceptualization of complex developmental trauma as a unitary construct that is appropriate for boys and girls.

#### Symptom‐specific gender differences in children

Clinically relevant differences in symptom expression emerged between girls and boys in childhood, particularly with regards to attachment‐related problem behaviors. Moderated networks and *t*‐tests revealed that girls tend to exhibit higher levels of over‐friendliness and affection‐ and attention‐seeking (*indiscriminate interpersonal behaviors*). These behaviors are consistent with Disinhibited Social Engagement Disorder (American Psychiatric Association, 2022; ZERO TO THREE, 2016), including precociousness (*pseudomature interpersonal behaviors*), and role‐reversal behaviors (i.e., parentification; Hooper et al., 2008), consistent with anxious‐preoccupied attachment (Bowlby, 1980; Lieberman & Zeanah, 1995). Girls may be more likely to exhibit such approach‐oriented behaviors following experiences of sexual abuse, which disproportionately impact girls (Fallon et al., 2022; Finkelhor et al., 2015; Moody et al., 2018; Stewart et al., 2020; Wamser‐Nanney & Cherry, 2018).

Boys, on the other hand, were found to exhibit more behaviors indicative of dismissive‐avoidant attachment (emotionally withdrawn, avoidant, and not reciprocating in social and emotional exchanges) and emotional dysregulation resulting in self‐harming behaviors. The *nonreciprocal interpersonal behaviors* exhibited by boys, if not conceptualized from an attachment theory perspective, may be erroneously diagnosed as oppositional defiance and as an early indication of antisocial traits (Ford et al., 2022). Such an interpersonal style may not only arise from violent victimization—which occurs more frequently in boys and can be related to higher levels of surgency and hyperactivity—but may be interpreted as intentional displays of opposition, contributing to future physical violence in high‐risk contexts (Fallon et al., 2022; Finkelhor et al., 2015; Moody et al., 2018).

#### Symptom‐specific gender differences in adolescents

We found adolescent girls to exhibit higher dysregulation symptomology, both emotionally and behaviorally. This contrasts the widely replicated finding, from studies with different measurement protocols and samples, that girls display more internalizing challenges and boys higher externalizing difficulties (Carragher et al., 2016), highlighting the different developmental trajectories adolescents may undergo after developmental trauma experiences. In tandem, the symptom profiles of adolescent girls are more elevated across the board in comparison to boys, including having more negative self‐concepts and higher rates of suicidality. Trauma experiences can have diverse, extensive impacts, and adolescent girls may be especially vulnerable to their effects. Additionally, girls are more likely to engage in maladaptive coping strategies such as rumination, which can exacerbate negatively valenced emotions and thoughts that stem from complex trauma (Martin & Hadwin, 2022). Conversely, boys may engage in alternative coping mechanisms, such as substance use, or use avoidance‐based strategies that suppress their experiences, leading to lower symptom reports by caregivers.

### Limitations and implications

The current study included several limitations. The analysis of gender differences was limited to boys and girls, and therefore did not include potential differential symptom expressions in gender‐diverse children and adolescents, due to pre‐existing limitations within the existing data collected by TFCP. That said, the rate of non‐binary gender identities with substantiated cases of maltreatment in the Ontario child welfare system is very low (<1%; Fallon et al., 2022), which would have precluded analyses within the present sample if such data were available. Other key sociodemographic data were unavailable in the study, including types and chronicity of trauma exposure, socioeconomic status, and racial/ethnic identity. Differential rates of exposure to types of maltreatment among boys and girls have been found (Fallon et al., 2022; Wamser‐Nanney & Cherry, 2018), which suggests a need for more specific gender‐based analyses accounting for trauma exposure. For example, Wamser‐Nanney and Cherry (2018) found gender‐differences in symptoms to persist among children exposed to child sexual abuse. Gender differences in symptom networks for specific types of maltreatment (e.g., neglect, physical, emotional, and sexual abuse) will be an important future direction. The present sample comes from rural catchment areas with predominantly white families. Also, ethnicity/race data were not systematically collected by the partnering agency. Hence, exploring the expression of complex trauma symptoms in diverse groups of children and adolescents is an important future direction. Additional moderators of interests, including care type and age into care, are also important considerations for future research examining complex trauma symptoms networks in maltreated children and youth.

The single‐informant methodology was another limitation, as the caregivers were the only source of information. In the future, it will be beneficial to collect data from various individuals who know the child/adolescent so that their symptoms can be evaluated from multiple perspectives and across various contexts. Given that the measures used in the present study were designed to capture the breadth of symptoms exhibited by children in out‐of‐home care and predated the proposed diagnostic criteria for Developmental Trauma Disorder and the ICD's Complex PTSD diagnosis, the present study did not examine classic PTSD symptoms, nor did it strictly align with recent frameworks of complex (developmental) trauma. Furthermore, having relatively high‐density regularized networks with few nodes (such as in the present study) increases power to detect differences with the network comparison permutation test (van Borkulo et al., 2023). However, estimating the networks with larger samples and more balanced group sizes will also be important in future replication studies, as simulation studies have shown this to increase power to detect differences in the global strength and network structure omnibus tests (van Borkulo et al., 2023). Finally, the cross‐sectional design did not allow for an analysis of causality and change over time, and future longitudinal studies should investigate the causes of specific symptom patterns in children and adolescents.

### Implications and conclusion

Despite the above limitations, several implications for clinical practice with children and adolescents who have experienced developmental trauma, and who are involved in the child welfare system, emerge from our results. The findings underscore the primacy of attachment insecurity and emotional dysregulation as being a central mechanism relevant to children's and adolescents' complex trauma symptomatology, consistent with the emergent conceptualization of complex developmental trauma (Cook et al., 2005; D’Andrea et al., 2012; McLaughlin et al., 2020; van der Kolk, 2005). Clinical assessment and practice should, therefore, intentionally consider the administration of validated, developmentally appropriate measures of complex trauma symptomatology that includes measures of attachment (such as the ACC/ACA). Further, the centrality of attachment‐specific symptoms and emotional dysregulation indicate that interventions that intentionally target improving attachment security and emotion regulation capacities may be particularly potent (Fried et al., 2017; Henry et al., 2022). This is consistent with a previous longitudinal study with the present child sample that found improvements in attachment security to prospectively predict improvements in psychosocial strengths 6 months later (Smith et al., 2023). Thus, caregiver‐oriented, whole‐family, and dyadic interventions that support caregivers and families with psychoeducation related to complex developmental trauma symptomatology, establishing safety, coaching sensitive and attuned responding and capacity for emotional co‐regulation, and relational healing are warranted. Several modalities have been developed with these principles in mind, including the Attachment, Regulation, and Competency framework (Blaustein & Kinniburgh, 2019), Biobehavioral Catch‐Up (Dozier et al., 2018), Child‐Parent Psychotherapy (Lieberman et al., 2015), Parent‐Child Interaction Therapy (Warren et al., 2022), and Trauma‐Focused Cognitive Behavior Therapy (Cohen et al., 2012). Considerations of how boys and girls tend to express symptoms will be an important addition to these interventions.

## AUTHOR CONTRIBUTIONS

**Jackson A. Smith**: Conceptualization; formal analysis; methodology; visualization; writing – original draft; writing – review & editing. **Jasmine Zhang**: Conceptualization; formal analysis; writing – original draft; writing – review & editing. **Alexey Urusov**: Conceptualization; writing – original draft; writing – review & editing. **Laura Colucci**: Conceptualization; writing – original draft. **Imogen Sloss**: Writing – original draft. **Lillian Eckert**: Writing – original draft. **Mary Price‐Cameron**: Conceptualization; data curation; writing – review & editing. **Dillon T.Browne**: Conceptualization; supervision; writing – original draft; writing – review & editing.

## CONFLICT OF INTEREST STATEMENT

The authors have declared they have no competing or potential conflicts of interest.Author contributions.

## Ethical considerations

This study was approved by the University of Waterloo Research Ethics Board (ORE #41024).

## Supporting information

Supporting Information S1

## Data Availability

The data that support the findings of this study are not publicly available due to privacy or ethical restrictions. Synthetic data have been generated and are available at https://doi.org/10.17605/OSF.IO/86WDN.
