# Supplementary material for: Exploring networks of complex developmental trauma symptomatology among children and adolescents involved in child welfare
Source: JCPP Adv. 2024 Mar 12;4(2):e12224. doi: 10.1002/jcv2.12224 (PMC11143960; doi:10.1002/jcv2.12224)
Supplement: Supplementary file 1 — Supporting Information S1 [file JCV2-4-e12224-s001.docx]

**Supplemental Material**

**Table S1**

***Normative, Borderline, and Clinical Ranges for the sub-scale raw scores of the Assessment Checklist for Children and Assessment Checklist for Adolescents.***

|  | **Boys** | | | **Girls** | | |
| --- | --- | --- | --- | --- | --- | --- |
| **Clinical Scales** | **Normal** | **Borderline** | **Clinical** | **Normal** | **Borderline** | **Clinical** |
| **Children – ACC Scales** |  |  |  |  |  |  |
| Abnormal Pain Response | 0-3 | 4 | 5-8 | 0-2 | 3-4 | 5-8 |
| Anxious Distrustful | 0-3 | 4-5 | 6-20 | 0-3 | 4-5 | 6-20 |
| Food Maintenance | 0-2 | 3-4 | 5-8 | 0-2 | 3-4 | 5-8 |
| Indiscriminate Interpersonal Behavior | 0-5 | 6-7 | 8-16 | 0-5 | 6-7 | 8-16 |
| Insecure Interpersonal Behavior | 0-3 | 4-5 | 6-28 | 0-3 | 4-5 | 6-28 |
| Non-Reciprocal Interpersonal Behavior | 0-3 | 4-5 | 6-24 | 0-3 | 4-5 | 6-24 |
| Pseudomature Interpersonal Behavior | 0-2 | 3-4 | 5-16 | 0-2 | 3-4 | 5-16 |
| Self Injury Index | 0-2 | 3-4 | 5-22 | 0-2 | 3-4 | 5-22 |
| Sexual Behaviors | 0 | 1-2 | 3-22 | 0-1 | 2-3 | 4-22 |
| Suicide Discourse | 0 | N/A | 1-14 | 0 | N/A | 1-14 |
| **Adolescents – ACA Scales** |  |  |  |  |  |  |
| Social Instability / Behavioral Dysregulation | 0-5 | 6-9 | 10-42 | 0-4 | 5-8 | 9-42 |
| Dissociation / Trauma Symptoms | 0-1 | 2-3 | 4-14 | 0-1 | 2-3 | 4-14 |
| Emotional Dysregulation / Distorted Social Cognition | 0-3 | 4-5 | 6-28 | 0-3 | 4-5 | 6-28 |
| Food Maintenance Behavior | 0-4 | 5-6 | 7-14 | 0-4 | 5-6 | 7-14 |
| Non-Reciprocal | 0-3 | 4-5 | 6-20 | 0-3 | 4-5 | 6-20 |
| Sexual Behaviors | 0 | 1-2 | 3-14 | 0 | 1-2 | 3-14 |
| Suicide Discourse | 0 | N/A | 1-12 | 0 | N/A | 1-12 |
| **Note.** The “Other Items” subscales of the ACC and ACA do not have established clinical cut-offs. | | | | | | |

**Table S2**

***Reliability of Assessment Checklist for Children (ACC) and Assessment Checklist for Children (ACA) Scales for the Full Sample, Boys, and Girls***

|  | **Full Sample** | **Boys** | **Girls** |
| --- | --- | --- | --- |
| **Children – ACC Scales** | *⍺* | *⍺* | *⍺* |
| Abnormal Pain Response | 0.39 | 0.62 | 0.33 |
| Anxious Distrustful | 0.68 | 0.72 | 0.66 |
| Food Maintenance | 0.61 | 0.72 | 0.70 |
| Indiscriminate | 0.71 | 0.66 | 0.77 |
| Insecure | 0.79 | 0.77 | 0.83 |
| Low Confidence | 0.73 | 0.75 | 0.78 |
| Negative Self-Image | 0.87 | 0.90 | 0.89 |
| Non-Reciprocal | 0.77 | 0.80 | 0.75 |
| Other Items | 0.50 | 0.59 | 0.47 |
| Pseudomature | 0.77 | 0.75 | 0.80 |
| Self Injury Total | 0.70 | 0.75 | 0.67 |
| Sexual | 0.68 | 0.73 | 0.72 |
| Suicide Discourse | 0.77 | 0.88 | 0.75 |
| **Adolescents - ACA Scales** | *⍺* | *⍺* | *⍺* |
| Behavioral Dysregulation | 0.89 | 0.87 | 0.89 |
| Dissociation and Trauma Symptoms | 0.57 | 0.59 | 0.56 |
| Emotional Dysregulation | 0.87 | 0.86 | 0.86 |
| Food Maintenance | 0.83 | 0.85 | 0.82 |
| Low Confidence | 0.78 | 0.81 | 0.76 |
| Negative Self-Image | 0.90 | 0.90 | 0.89 |
| Non-Reciprocal Interpersonal Behaviors | 0.77 | 0.81 | 0.72 |
| Other Items | 0.76 | 0.77 | 0.74 |
| Sexual Behaviors | 0.76 | 0.77 | 0.73 |
| Suicide Discourse | 0.86 | 0.81 | 0.86 |

**Table S3**

***Weight Matrices of Child Networks for Boys and Girls***

| ACC Subscale | ACC1 | ACC2 | ACC3 | ACC4 | ACC5 | ACC6 | ACC7 | ACC8 | ACC9 | ACC10 | ACC11 |
| --- | --- | --- | --- | --- | --- | --- | --- | --- | --- | --- | --- |
| **Boys** |  |  |  |  |  |  |  |  |  |  |  |
| ACC1 | - |  |  |  |  |  |  |  |  |  |  |
| ACC2 | 0.09 | - |  |  |  |  |  |  |  |  |  |
| ACC3 | 0.14 | 0.33 | - |  |  |  |  |  |  |  |  |
| ACC4 | 0.10 | 0.15 | 0.02 | - |  |  |  |  |  |  |  |
| ACC5 | 0.05 | 0.13 | 0.19 | - | - |  |  |  |  |  |  |
| ACC6 | - | - | 0.10 | 0.12 | 0.40 | - |  |  |  |  |  |
| ACC7 | 0.01 | - | 0.11 | 0.21 | 0.01 | - | - |  |  |  |  |
| ACC8 | 0.16 | - | 0.07 | 0.12 | - | 0.07 | 0.21 | - |  |  |  |
| ACC9 | - | 0.08 | - | - | - | - | -0.05 | 0.13 | - |  |  |
| ACC10 | - | - | - | - | 0.40 | 0.01 | - | - | 0.22 | - |  |
| ACC11 | -0.02 | -0.11 | 0.09 | - | 0.25 | - | - | 0.07 | - | 0.27 | - |
| **Girls** |  |  |  |  |  |  |  |  |  |  |  |
| ACC1 | - |  |  |  |  |  |  |  |  |  |  |
| ACC2 | - | - |  |  |  |  |  |  |  |  |  |
| ACC3 | 0.03 | 0.36 | - |  |  |  |  |  |  |  |  |
| ACC4 | 0.22 | 0.30 | - | - |  |  |  |  |  |  |  |
| ACC5 | 0.11 | 0.18 | 0.08 | - | - |  |  |  |  |  |  |
| ACC6 | 0.04 | - | 0.01 | 0.05 | 0.30 | - |  |  |  |  |  |
| ACC7 | - | - | 0.14 | 0.15 | 0.05 | 0.04 | - |  |  |  |  |
| ACC8 | - | - | 0.03 | 0.07 | - | - | - | - |  |  |  |
| ACC9 | - | - | - | - | - | - | - | 0.18 | - |  |  |
| ACC10 | - | - | - | - | 0.34 | - | 0.01 | 0.02 | 0.26 | - |  |
| ACC11 | - | - | 0.22 | 0.03 | 0.20 | 0.10 | - | 0.05 | 0.04 | 0.19 | - |
| *Note.* ACC1: Sexual Behaviors. ACC2: Pseudomature Interpersonal Behaviors. ACC3: Non-Reciprocal Interpersonal Behaviors. ACC4: Indiscriminate Interpersonal Behaviors. ACC5: Insecure Interpersonal Behaviors. ACC6: Anxious-Distrustful. ACC7: Food Maintenance. ACC8: Self-Injury. ACC9: Suicide Discourse. ACC10: Negative Self-Image. ACC11: Low Confidence. | | | | | | | | | | | |

**Table S4**

***Weight Matrices of Adolescent Networks for Boys and Girls***

| ACA Subscale | ACA1 | ACA2 | ACA3 | ACA4 | ACA5 | ACA6 | ACA7 | ACA8 | ACA9 | ACA10 |
| --- | --- | --- | --- | --- | --- | --- | --- | --- | --- | --- |
| **Boys** |  |  |  |  |  |  |  |  |  |  |
| ACA1 | - |  |  |  |  |  |  |  |  |  |
| ACA2 | 0.08 | - |  |  |  |  |  |  |  |  |
| ACA3 | - | 0.31 | - |  |  |  |  |  |  |  |
| ACA4 | - | - | 0.12 | - |  |  |  |  |  |  |
| ACA5 | 0.06 | 0.05 | 0.07 | - | - |  |  |  |  |  |
| ACA6 | - | 0.16 | - | - | 0.11 | - |  |  |  |  |
| ACA7 | - | - | 0.20 | 0.18 | 0.10 | 0.20 | - |  |  |  |
| ACA8 | 0.15 | 0.22 | 0.26 | 0.26 | - | 0.02 | - | - |  |  |
| ACA9 | - | - | 0.12 | 0.09 | - | - | 0.10 | 0.21 | - |  |
| ACA10 | 0.15 | - | 0.12 | - | 0.18 | - | - | 0.11 | 0.34 | - |
| **Girls** |  |  |  |  |  |  |  |  |  |  |
| ACA1 | - |  |  |  |  |  |  |  |  |  |
| ACA2 | 0.09 | - |  |  |  |  |  |  |  |  |
| ACA3 | 0.12 | 0.21 | - |  |  |  |  |  |  |  |
| ACA4 | - | 0.10 | 0.11 | - |  |  |  |  |  |  |
| ACA5 | 0.10 | 0.23 | - | - | - |  |  |  |  |  |
| ACA6 | - | 0.24 | - | 0.04 | 0.12 | - |  |  |  |  |
| ACA7 | - | - | 0.24 | 0.20 | - | 0.11 | - |  |  |  |
| ACA8 | 0.10 | 0.16 | 0.28 | 0.15 | 0.01 | 0.05 | 0.18 | - |  |  |
| ACA9 | - | -0.01 | 0.21 | - | - | -0.10 | - | 0.33 | - |  |
| ACA10 | 0.13 | - | - | - | 0.05 | - | - | 0.06 | 0.44 | - |

**Additional Centrality Measures of ACC and ACA Networks**

Standardized betweenness and closeness centrality measures for gender-specific networks were somewhat unstable (Table 4): Betweenness was low for the boy network (CS(cor = 0.70) = 0.21) and closeness was low for the girl network (CS(cor = 0.70) = 0.13). Hence, these measures were not interpreted. For the boy network, Insecure interpersonal behaviors showed the strongest closeness and expected influence centrality (with standardized estimates of 1.64 and 2.44, respectively), indicating that this subscale is well-connected to other nodes in the network, both directly and indirectly. Non-reciprocal behaviors emerged as the next most influential node (closeness = 1.27, expected influence = 1.03), followed by pseudomature behaviors (closeness = 0.53, expected influence = 0.59), and negative self-image (closeness = 0.61, expected influence = 0.47). For girls, the pseudomature behaviors node had strong direct associations with other nodes and cast a strong cumulative influence within the network (betweenness = 1.65, expected influence = 0.59), followed by Insecure behaviors (betweenness = 1.10, expected influence = 1.68).

Standardized centrality measures of betweenness for gender-specific networks showed low stability, as indicated by CS coefficients (boy network: CS(cor = 0.70) = 0.21, girl network: CS(cor = 0.70) = 0.13). Hence, these measures were not interpreted. In the boy network, emotion dysregulation appeared to be well-connected with other nodes and was cumulatively influential in the network (closeness = 1.64, expected influence = 1.46). Other Items was also an influential node (closeness = 1.32, expected influence = 1.51). For the girl network, Other Items emerged as the most influential node (closeness = 1.79, expected influence = 1.95), followed by Emotion Dysregulation (closeness = 1.34, expected influence = 1.16) and Behavioral Dysregulation (closeness = 0.54, expected influence = 0.59) and Negative Self-Image (closeness = 0.45, expected influence = 0.48).

**Table S5**

***Node Centrality Statistics for All Network Models***

|  | **Betweenness** | | **Closeness** | | **Strength** | | **Expected Influence** | |
| --- | --- | --- | --- | --- | --- | --- | --- | --- |
| **Children – ACC Scales** | **Boy** | **Girl** | **Boy** | **Girl** | **Boy** | **Girl** | **Boy** | **Girl** |
| Anxious-Distrustful | - | -1.10 | 0.30 | - | -0.60 | -0.71 | -0.47 | -0.52 |
| Food Maintenance | - | -1.10 | -0.81 | - | -0.61 | -1.41 | -0.48 | -1.29 |
| Indiscriminate | - | 1.10 | -0.41 | - | -0.28 | 0.47 | -0.14 | 0.79 |
| Insecure | - | 1.10 | 1.64 | - | 2.31 | 1.27 | 2.44 | 1.68 |
| Low Confidence | - | -0.37 | 0.26 | - | 0.27 | 0.91 | -0.33 | 0.58 |
| Negative Self-Image | - | 0.37 | 0.61 | - | 0.23 | 0.18 | 0.36 | 0.47 |
| Non-Reciprocal | - | 0.55 | 1.27 | - | 0.90 | 0.73 | 1.03 | 0.72 |
| Pseudomature | - | 1.65 | 0.53 | - | 0.31 | 1.30 | -0.30 | 0.59 |
| Self-Injury | - | -0.73 | -0.85 | - | -0.42 | -1.06 | -0.28 | -1.33 |
| Sexual Behavior | - | -1.10 | -1.06 | - | -0.61 | -1.08 | -0.47 | -0.93 |
| Suicide Discourse | - | -0.37 | -1.48 | - | -1.50 | -0.59 | -1.37 | -0.74 |
| **Adolescents – ACA Scales** | **Boy** | **Girl** | **Boy** | **Girl** | **Boy** | **Girl** | **Boy** | **Girl** |
| Behavioral Dysregulation | - | - | 0.76 | 0.54 | 0.65 | 0.59 | 0.65 | 0.59 |
| Dissociation & Trauma Symptoms | - | - | -0.81 | -0.73 | -0.89 | -0.49 | -0.89 | -0.49 |
| Emotion Dysregulation | - | - | 1.64 | 1.34 | 1.42 | 1.16 | 1.42 | 1.16 |
| Food Maintenance | - | - | -1.03 | -0.89 | -0.74 | -0.85 | -0.74 | -0.85 |
| Low Confidence | - | - | 0.09 | -0.19 | 0.44 | -0.39 | 0.44 | -0.39 |
| Negative Self-Image | - | - | 0.27 | 0.45 | 0.15 | 0.48 | 0.15 | 0.48 |
| Non-Reciprocal | - | - | -1.07 | -0.99 | -1.22 | -0.71 | -1.22 | -0.71 |
| Other Items | - | - | 1.32 | 1.79 | 1.51 | 1.95 | 1.51 | 1.95 |
| Sexual Behavior | - | - | -1.01 | -1.04 | -1.09 | -1.11 | -1.09 | -1.11 |
| Suicide Discourse | - | - | -0.16 | -0.29 | -0.23 | -0.63 | -0.23 | -0.63 |
| *Note.* Betweenness centrality for boys and closeness centrality for girls were excluded due to low CS coefficients (i.e., CS(cor = 0.70) < 0.25). | | | | | | | | |

**Table S6**

***Correlation-Stability (CS) Coefficients of Network Models***

|  | **Correlation-stability (CS) coefficients** | |
| --- | --- | --- |
| **Centrality measure** | **Boys** | **Girls** |
| **Children** |  |  |
| Betweenness | 0.21 | 0.28 |
| Closeness | 0.36 | 0.13 |
| Strength | 0.59 | 0.36 |
| Expected Influence | 0.59 | 0.36 |
| **Adolescents** |  |  |
| Betweenness | 0.13 | 0.21 |
| Closeness | 0.44 | 0.52 |
| Strength | 0.51 | 0.60 |
| Expected Influence | 0.51 | 0.60 |

**Figure S1**

***Centrality Indices of Child Networks for Boys and Girls***

**
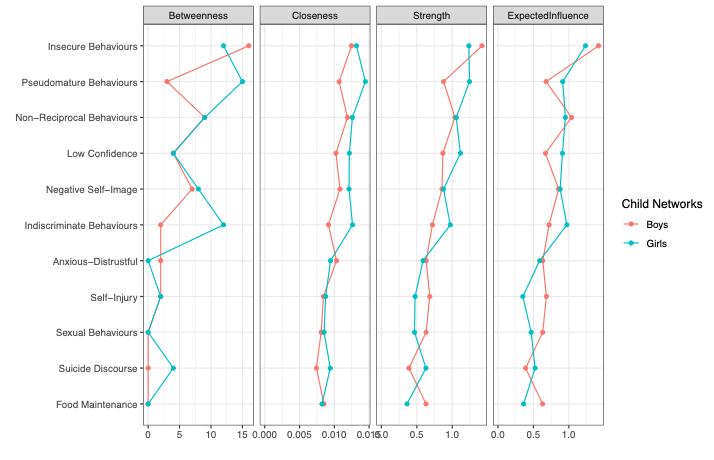
**

**Figure S2**

***Centrality Indices of Adolescent Networks for Boys and Girls***

**
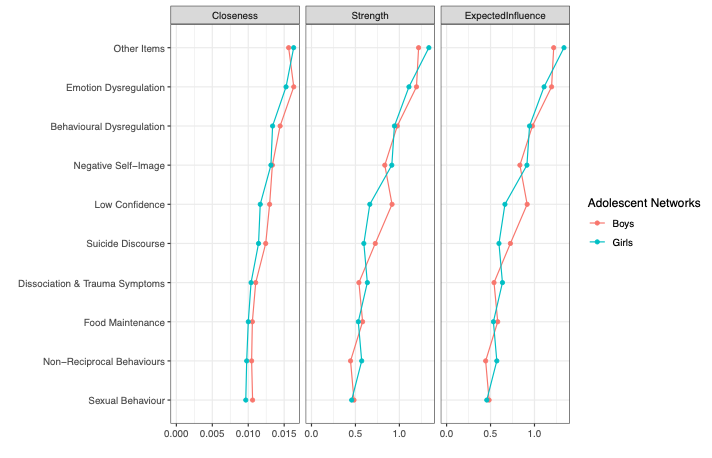
**

**Figure S3**

***Edge Weight Stability for Child Networks***


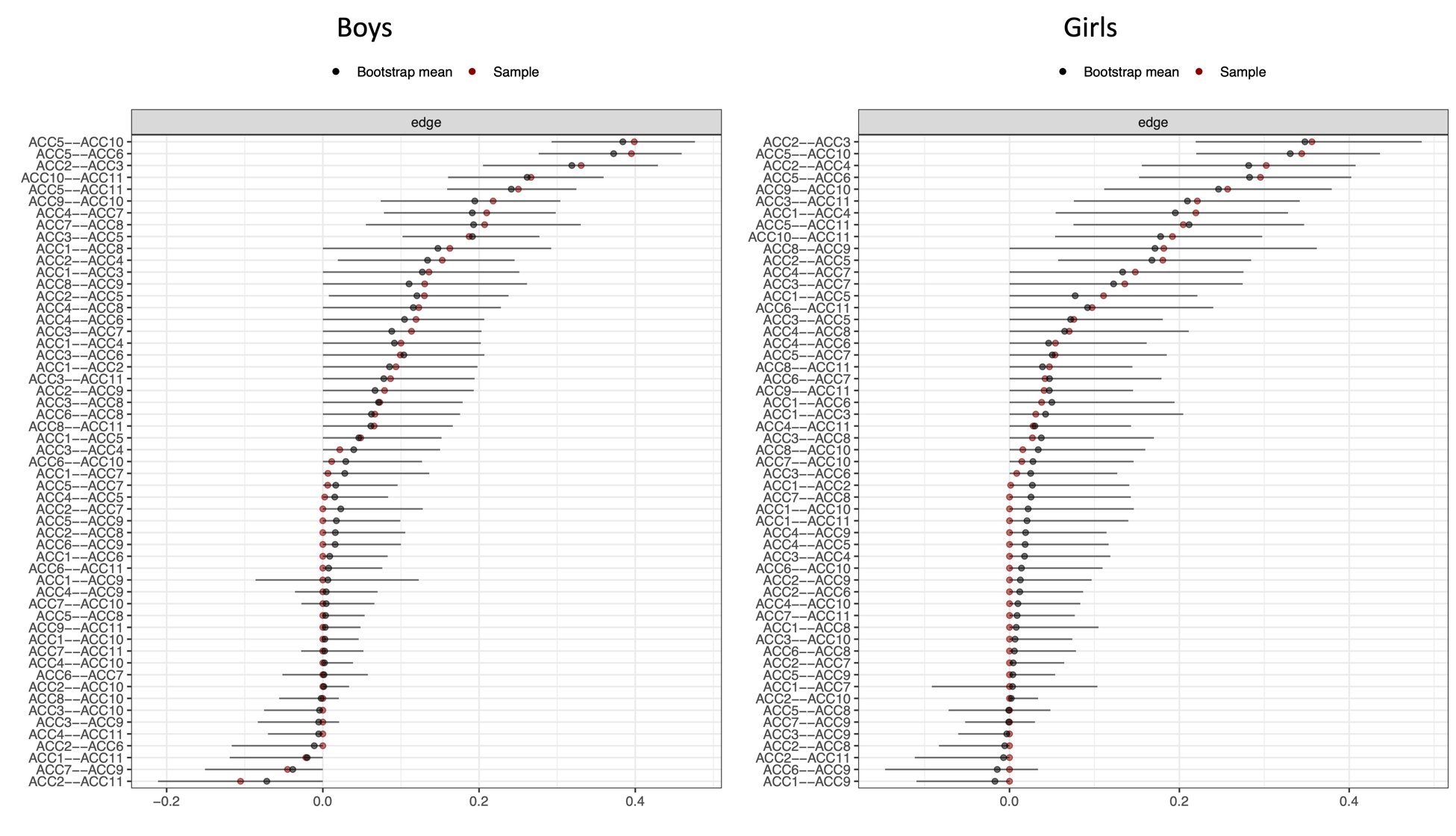


*Note.* The left panel displays results for the boy network, and the right panel displays results for girl network. The red line shows edge weights, and the grey lines display 95% confidence intervals based on 2500 bootstraps. Narrower confidence intervals that remain close to the edge weight value suggest high stability. Overlapping intervals indicate that edge weights are unlikely to significantly differ from one-another.

**Figure S4**

***Edge Weight Stability for Adolescent Networks***

**
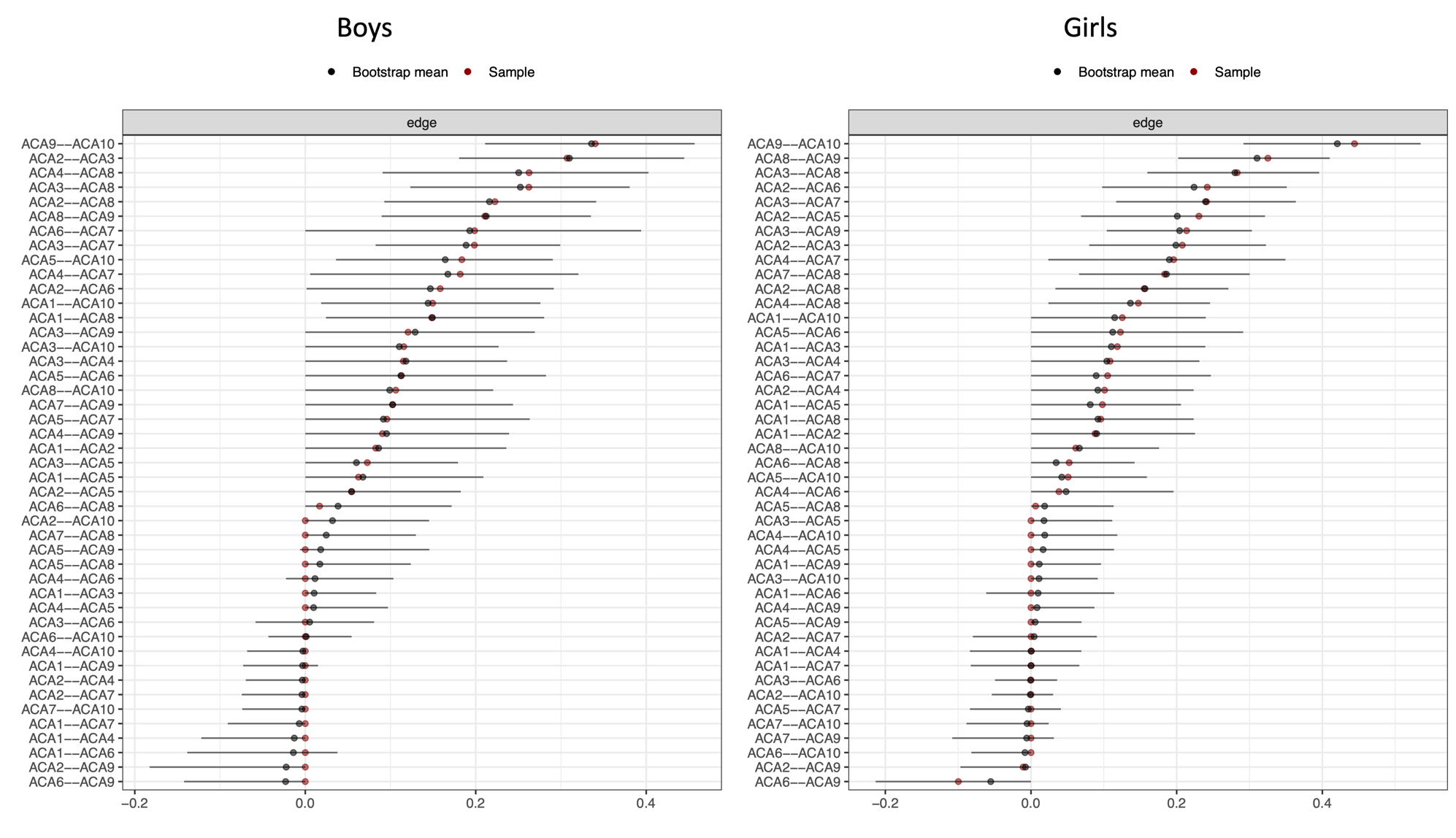
**

*Note.* The left panel displays results for the boy network, and the right panel displays results for girl network. The red line shows edge weights, and the grey lines display 95% confidence intervals based on 2500 bootstraps. Narrower confidence intervals that remain close to the edge weight value suggest high stability. Overlapping intervals indicate that edge weights are unlikely to significantly differ from one-another.
